# Poly[hexa­aqua­tri-μ-malonato-didysprosium(III)]

**DOI:** 10.1107/S1600536808015961

**Published:** 2008-06-07

**Authors:** Zhan-Qiang Fang, Rong-Hua Zeng, Zhao-Feng Song, Mei Yang

**Affiliations:** a School of Chemistry and Environment, South China Normal University, Guangzhou 510006, People’s Republic of China

## Abstract

The title compound, [Dy_2_(C_3_H_2_O_4_)_3_(H_2_O)_6_]_*n*_, forms a coordination polymeric structure comprising hydrated dysprosium ions and malonate ligands. In the asymmetric unit, there are one dysprosium ion, one and a half malonate ligands, and three water mol­ecules. Each Dy^III^ atom is coordinated by six O atoms from four malonate ligands and by three water mol­ecules, and displays a tricapped trigonal–prismatic coordination geometry. The malonate ligands adopt two types of coordination mode, linking dysprosium centres to form a three-dimensional coordination polymer. The extensive network of hydrogen bonds in this polymer enhances the structural stability.

## Related literature

For related literature, see: Iglesias *et al.* (2003 ▶); Kim *et al.* (2003 ▶); Moulton & Zaworotko (2001 ▶). 
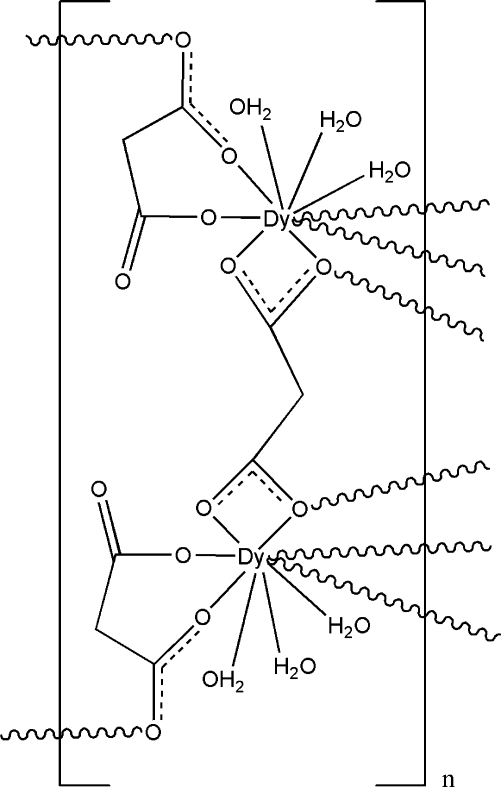


## Experimental

### 

#### Crystal data

[Dy_2_(C_3_H_2_O_4_)_3_(H_2_O)_6_]
*M* *_r_* = 739.23
Monoclinic, 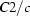

*a* = 17.1805 (2) Å
*b* = 12.3124 (1) Å
*c* = 11.1541 (1) Å
β = 127.52 (2)°
*V* = 1871.4 (5) Å^3^
*Z* = 4
Mo *K*α radiation
μ = 8.02 mm^−1^
*T* = 296 (2) K
0.11 × 0.10 × 0.08 mm

#### Data collection

Bruker APEXII area-detector diffractometer
Absorption correction: multi-scan (*APEX2*; Bruker, 2004 ▶) *T*_min_ = 0.435, *T*_max_ = 0.529
10051 measured reflections
2136 independent reflections
2001 reflections with *I* > 2σ(*I*)
*R*_int_ = 0.023

#### Refinement

*R*[*F* ^2^ > 2σ(*F* ^2^)] = 0.019
*wR*(*F* ^2^) = 0.053
*S* = 1.07
2136 reflections
132 parameters
10 restraints
H-atom parameters constrained
Δρ_max_ = 0.91 e Å^−3^
Δρ_min_ = −0.51 e Å^−3^

### 

Data collection: *APEX2* (Bruker, 2004 ▶); cell refinement: *APEX2*; data reduction: *APEX2*; program(s) used to solve structure: *SHELXS97* (Sheldrick, 2008 ▶); program(s) used to refine structure: *SHELXL97* (Sheldrick, 2008 ▶); molecular graphics: *PLATON* (Spek, 2003 ▶) and *SHELXTL* (Sheldrick, 2008 ▶); software used to prepare material for publication: *SHELXL97*.

## Supplementary Material

Crystal structure: contains datablocks I, global. DOI: 10.1107/S1600536808015961/dn2344sup1.cif

Structure factors: contains datablocks I. DOI: 10.1107/S1600536808015961/dn2344Isup2.hkl

Additional supplementary materials:  crystallographic information; 3D view; checkCIF report

## Figures and Tables

**Table 1:** Hydrogen-bond geometry (Å, °)

*D*—H⋯*A*	*D*—H	H⋯*A*	*D*⋯*A*	*D*—H⋯*A*
O1*W*—H1*W*⋯O5^i^	0.82	2.04	2.854 (4)	172
O1*W*—H2*W*⋯O3^ii^	0.81	1.94	2.729 (4)	165
O2*W*—H3*W*⋯O3^iii^	0.82	1.95	2.761 (4)	170
O3*W*—H6*W*⋯O4^iii^	0.81	2.02	2.802 (4)	160
O3*W*—H6*W*⋯O3^iii^	0.81	2.59	3.291 (4)	144
O3*W*—H5*W*⋯O2^iv^	0.81	1.96	2.738 (4)	161

Symmetry codes: (i) 
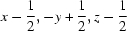
; (ii) 
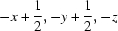
; (iii) 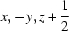; (iv) 
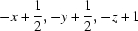
.

**Fig. 1.**
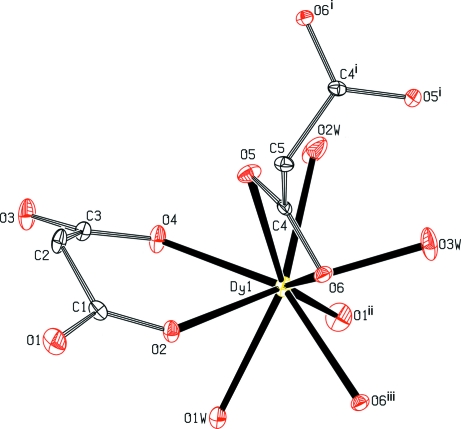
The molecular structure showing the atomic-numbering scheme. Displacement ellipsoids drawn at the 30% probability level. H atoms have been omitted for clarity. [Symmetry codes: (i) 1-x, y, 3/2-y; (ii) 1/2-x, y-1/2, 1/2-z; (iii) 1/2-x, 1/2-y, 1-z]

**Fig. 2.**
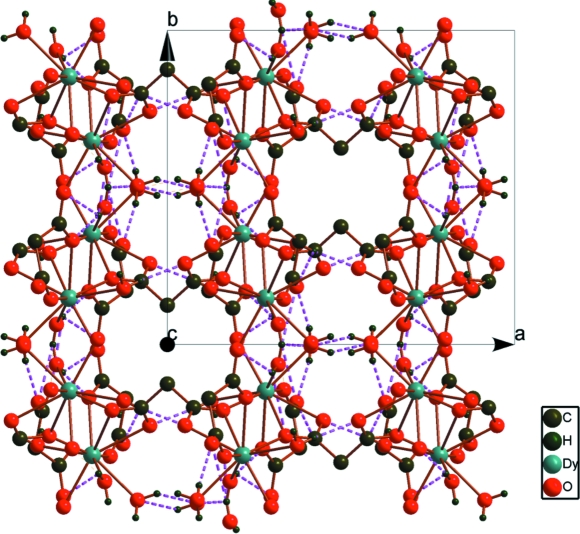
The molecular packing showing the intra/intermolecular hydrogen bonding interactions as broken lines.

## supplementary crystallographic information

### Comment

Molecular self-assembly of supramolecular architectures has received much attention during recent decades (Kim *et al.*, 2003; Iglesias *et al.*, 2003; Moulton & Zaworotko, 2001). The structures and properties of such systems depend on the coordination and geometric preferences of both the central metals ions and bridging building blocks as well as the influence of weaker non-covalent interactions, such as hydrogen bonds and π-π stacking interactions. Recently, we obtained the title compound, (I), by the hydrothermal reaction of Dy(NO_3_)_3_ with malonic acid in alkaline aqueous solution.

As illustrated in Fig. 1, in the asymmetric unit of complex (I), each Dy^III^ centre is coordinated by six carboxyl O atoms from four malonate ligands, and three water molecules. The two unique malonate ligands act as two types of chelating and bridging modes: one lies on an inversion centre and uses each carboxylate group to bond to two Dy^III^ ions; one uses three carboxyl O atoms to coordinate to two Dy^III^ ions involving a six-membered chelate ring. The adjacent Dy···Dy separations are 4.303 (3), 6.600 (1) and 6.982 (2) Å respectively. The ligands link dysprosium centres to form a three-dimensional coordination polymer which is also stabilized by the extensive network of hydrogen bonding interactions (Fig. 2; Table 1).

### Experimental

A mixture of Dy(NO_3_)_3_ (0.1 mmol), malonato acid (0.15 mmol), NaOH (0.1 mmol), water (10 ml) was stirred vigorously for 20 min and then sealed in a Teflon-lined stainless-steel autoclave (20 ml, capacity). The autoclave was heated to and maintained at 433 K for 7 days, and then cooled to room temperature at 5 K h^-1^ to obtain the colorless block crystals.

### Refinement

Water H atoms were tentatively located in difference Fourier maps and were refined with distance restraints of O–H = 0.82 Å and H···H = 1.30 Å, and with *U*_iso_(H) = 1.5 *U*_eq_(O), and then were treated as riding mode. Carbon-bound H atoms were placed at calculated positions and were treated as riding on the parent C atoms with C—H = 0.97 Å, and with *U*_iso_(H) = 1.2 *U*_eq_(C).

### Crystal data

**Table:**

[Dy_2_(C_3_H_2_O_4_)_3_(H_2_O)_6_]	*F*_000_ = 1392
*M**_r_* = 739.23	*D*_x_ = 2.624 Mg m^−^^3^
Monoclinic, *C*2/*c*	Mo *K*α radiation λ = 0.71073 Å
Hall symbol: -C 2yc	Cell parameters from 6377 reflections
*a* = 17.1805 (2) Å	θ = 1.7–28.0º
*b* = 12.3124 (1) Å	µ = 8.02 mm^−^^1^
*c* = 11.1541 (1) Å	*T* = 296 (2) K
β = 127.52 (2)º	Block, colorless
*V* = 1871.4 (5) Å^3^	0.11 × 0.10 × 0.08 mm
*Z* = 4	

### Data collection

**Table:**

Bruker APEXII area-detector diffractometer	2136 independent reflections
Radiation source: fine-focus sealed tube	2001 reflections with *I* > 2σ(*I*)
Monochromator: graphite	*R*_int_ = 0.023
*T* = 296(2) K	θ_max_ = 27.5º
φ and ω scans	θ_min_ = 2.2º
Absorption correction: multi-scan(APEX2; Bruker, 2004)	*h* = −22→20
*T*_min_ = 0.435, *T*_max_ = 0.529	*k* = −15→15
10051 measured reflections	*l* = −12→14

### Refinement

**Table:**

Refinement on *F*^2^	Secondary atom site location: difference Fourier map
Least-squares matrix: full	Hydrogen site location: inferred from neighbouring sites
*R*[*F*^2^ > 2σ(*F*^2^)] = 0.020	H-atom parameters constrained
*wR*(*F*^2^) = 0.053	*w* = 1/[σ^2^(*F*_o_^2^) + (0.0241*P*)^2^ + 12.727*P*] where *P* = (*F*_o_^2^ + 2*F*_c_^2^)/3
*S* = 1.07	(Δ/σ)_max_ = 0.001
2136 reflections	Δρ_max_ = 0.91 e Å^−^^3^
132 parameters	Δρ_min_ = −0.51 e Å^−^^3^
10 restraints	Extinction correction: none
Primary atom site location: structure-invariant direct methods	

### Special details

**Table:**

Geometry. All e.s.d.'s (except the e.s.d. in the dihedral angle between two l.s. planes) are estimated using the full covariance matrix. The cell e.s.d.'s are taken into account individually in the estimation of e.s.d.'s in distances, angles and torsion angles; correlations between e.s.d.'s in cell parameters are only used when they are defined by crystal symmetry. An approximate (isotropic) treatment of cell e.s.d.'s is used for estimating e.s.d.'s involving l.s. planes.
Refinement. Refinement of *F*^2^ against ALL reflections. The weighted *R*-factor *wR* and goodness of fit *S* are based on *F*^2^, conventional *R*-factors *R* are based on *F*, with *F* set to zero for negative *F*^2^. The threshold expression of *F*^2^ > σ(*F*^2^) is used only for calculating *R*-factors(gt) *etc*. and is not relevant to the choice of reflections for refinement. *R*-factors based on *F*^2^ are statistically about twice as large as those based on *F*, and *R*- factors based on ALL data will be even larger.

### Fractional atomic coordinates and isotropic or equivalent isotropic displacement parameters (Å^2^)

**Table:**

	*x*	*y*	*z*	*U*_iso_*/*U*_eq_	Occ. (<1)
C1	0.3116 (3)	0.3839 (3)	0.2419 (4)	0.0139 (7)	
C2	0.3805 (3)	0.3285 (3)	0.2198 (5)	0.0174 (7)	
H2A	0.4462	0.3325	0.3148	0.021*	
H2B	0.3808	0.3704	0.1464	0.021*	
C3	0.3607 (3)	0.2110 (3)	0.1686 (4)	0.0137 (7)	
C4	0.4246 (2)	0.2993 (3)	0.6243 (4)	0.0119 (7)	
C5	0.5000	0.3747 (4)	0.7500	0.0135 (10)	
H5A	0.4703	0.4205	0.7829	0.016*	0.50
H5B	0.5297	0.4205	0.7171	0.016*	0.50
Dy1	0.283235 (12)	0.148077 (13)	0.379865 (19)	0.01461 (7)	
O1	0.2989 (2)	0.4832 (2)	0.2144 (4)	0.0268 (6)	
O2	0.2741 (2)	0.3315 (2)	0.2918 (3)	0.0165 (5)	
O3	0.3717 (2)	0.1844 (2)	0.0723 (3)	0.0256 (6)	
O4	0.3355 (2)	0.14437 (19)	0.2259 (3)	0.0200 (6)	
O5	0.44917 (18)	0.2424 (2)	0.5591 (3)	0.0192 (5)	
O6	0.34060 (17)	0.2909 (2)	0.5918 (3)	0.0144 (5)	
O1W	0.1257 (2)	0.1789 (2)	0.1179 (3)	0.0226 (6)	
H1W	0.0785	0.2029	0.1100	0.034*	
H2W	0.1369	0.2230	0.0756	0.034*	
O2W	0.4141 (3)	0.0048 (3)	0.4897 (5)	0.0435 (10)	
H3W	0.4088	−0.0512	0.5238	0.065*	
H4W	0.4700	0.0253	0.5538	0.065*	
O3W	0.3194 (2)	0.0640 (2)	0.6116 (4)	0.0322 (7)	
H6W	0.3294	−0.0003	0.6333	0.048*	
H5W	0.3019	0.0889	0.6593	0.048*	

### Atomic displacement parameters (Å^2^)

**Table:**

	*U*^11^	*U*^22^	*U*^33^	*U*^12^	*U*^13^	*U*^23^
C1	0.0179 (17)	0.0087 (15)	0.0121 (17)	0.0002 (13)	0.0077 (15)	−0.0012 (13)
C2	0.0267 (19)	0.0119 (16)	0.024 (2)	−0.0038 (14)	0.0211 (18)	−0.0010 (14)
C3	0.0166 (17)	0.0121 (16)	0.0163 (18)	−0.0007 (13)	0.0119 (15)	−0.0004 (13)
C4	0.0099 (15)	0.0139 (16)	0.0082 (16)	−0.0002 (12)	0.0037 (14)	0.0026 (13)
C5	0.010 (2)	0.011 (2)	0.014 (2)	0.000	0.005 (2)	0.000
Dy1	0.01901 (10)	0.01230 (10)	0.01759 (11)	−0.00036 (6)	0.01376 (8)	−0.00016 (6)
O1	0.0366 (17)	0.0102 (12)	0.0372 (18)	0.0042 (11)	0.0244 (15)	0.0062 (12)
O2	0.0249 (14)	0.0116 (12)	0.0203 (14)	0.0034 (10)	0.0175 (12)	0.0027 (10)
O3	0.0474 (18)	0.0187 (14)	0.0288 (16)	−0.0005 (13)	0.0326 (16)	−0.0014 (12)
O4	0.0366 (16)	0.0100 (12)	0.0276 (16)	−0.0013 (10)	0.0270 (14)	−0.0012 (10)
O5	0.0137 (12)	0.0264 (14)	0.0183 (13)	−0.0008 (10)	0.0102 (11)	−0.0068 (11)
O6	0.0104 (11)	0.0196 (13)	0.0131 (12)	−0.0015 (9)	0.0072 (10)	−0.0016 (10)
O1W	0.0206 (14)	0.0300 (15)	0.0198 (15)	−0.0003 (12)	0.0137 (12)	0.0065 (12)
O2W	0.0431 (19)	0.0277 (17)	0.085 (3)	0.0166 (15)	0.052 (2)	0.0281 (18)
O3W	0.063 (2)	0.0176 (14)	0.0415 (19)	0.0177 (14)	0.0450 (18)	0.0154 (13)

### Geometric parameters (Å, °)

**Table:**

C1—O1	1.247 (4)	Dy1—O4	2.375 (3)
C1—O2	1.256 (4)	Dy1—O2	2.430 (2)
C1—C2	1.512 (5)	Dy1—O6^iii^	2.452 (2)
C2—C3	1.516 (5)	Dy1—O3W	2.487 (3)
C2—H2A	0.9700	Dy1—O2W	2.513 (3)
C2—H2B	0.9700	Dy1—O1W	2.524 (3)
C3—O3	1.243 (4)	Dy1—O5	2.555 (3)
C3—O4	1.266 (4)	Dy1—O6	2.610 (2)
C4—O5	1.254 (4)	O1W—H1W	0.8155
C4—O6	1.260 (4)	O1W—H2W	0.8146
C4—C5	1.514 (4)	O2W—H3W	0.8184
C5—C4^i^	1.514 (4)	O2W—H4W	0.8133
C5—H5A	0.9700	O3W—H6W	0.8149
C5—H5B	0.9700	O3W—H5W	0.8144
Dy1—O1^ii^	2.326 (3)		
			
O1—C1—O2	123.5 (3)	O4—Dy1—O1W	77.10 (10)
O1—C1—C2	116.0 (3)	O2—Dy1—O1W	68.42 (9)
O2—C1—C2	120.4 (3)	O6^iii^—Dy1—O1W	72.58 (9)
C1—C2—C3	118.3 (3)	O3W—Dy1—O1W	132.74 (10)
C1—C2—H2A	107.7	O2W—Dy1—O1W	132.85 (12)
C3—C2—H2A	107.7	O1^ii^—Dy1—O5	146.20 (10)
C1—C2—H2B	107.7	O4—Dy1—O5	80.87 (9)
C3—C2—H2B	107.7	O2—Dy1—O5	70.15 (9)
H2A—C2—H2B	107.1	O6^iii^—Dy1—O5	113.70 (8)
O3—C3—O4	123.0 (3)	O3W—Dy1—O5	85.58 (10)
O3—C3—C2	117.3 (3)	O2W—Dy1—O5	72.37 (10)
O4—C3—C2	119.7 (3)	O1W—Dy1—O5	137.36 (9)
O5—C4—O6	121.2 (3)	O1^ii^—Dy1—O6	141.99 (9)
O5—C4—C5	118.7 (3)	O4—Dy1—O6	124.57 (8)
O6—C4—C5	120.1 (3)	O2—Dy1—O6	68.62 (8)
C4—C5—C4^i^	104.4 (4)	O6^iii^—Dy1—O6	63.60 (9)
C4—C5—H5A	110.9	O3W—Dy1—O6	67.78 (9)
C4^i^—C5—H5A	110.9	O2W—Dy1—O6	107.42 (11)
C4—C5—H5B	110.9	O1W—Dy1—O6	119.61 (9)
C4^i^—C5—H5B	110.9	O5—Dy1—O6	50.15 (8)
H5A—C5—H5B	108.9	C1—O1—Dy1^iv^	159.0 (3)
O1^ii^—Dy1—O4	92.80 (10)	C1—O2—Dy1	137.0 (2)
O1^ii^—Dy1—O2	139.15 (10)	C3—O4—Dy1	138.4 (2)
O4—Dy1—O2	71.67 (8)	C4—O5—Dy1	95.7 (2)
O1^ii^—Dy1—O6^iii^	89.46 (9)	C4—O6—Dy1^iii^	150.4 (2)
O4—Dy1—O6^iii^	147.09 (9)	C4—O6—Dy1	92.9 (2)
O2—Dy1—O6^iii^	85.38 (8)	Dy1^iii^—O6—Dy1	116.40 (9)
O1^ii^—Dy1—O3W	78.76 (11)	Dy1—O1W—H1W	118.3
O4—Dy1—O3W	141.16 (9)	Dy1—O1W—H2W	107.9
O2—Dy1—O3W	136.14 (9)	H1W—O1W—H2W	105.4
O6^iii^—Dy1—O3W	71.36 (9)	Dy1—O2W—H3W	119.8
O1^ii^—Dy1—O2W	73.98 (11)	Dy1—O2W—H4W	115.9
O4—Dy1—O2W	73.66 (10)	H3W—O2W—H4W	105.1
O2—Dy1—O2W	131.94 (9)	Dy1—O3W—H6W	126.0
O6^iii^—Dy1—O2W	137.86 (9)	Dy1—O3W—H5W	124.4
O3W—Dy1—O2W	67.55 (10)	H6W—O3W—H5W	105.5
O1^ii^—Dy1—O1W	71.37 (10)		

Symmetry codes: (i) −*x*+1, *y*, −*z*+3/2; (ii) −*x*+1/2, *y*−1/2, −*z*+1/2; (iii) −*x*+1/2, −*y*+1/2, −*z*+1; (iv) −*x*+1/2, *y*+1/2, −*z*+1/2.

### Hydrogen-bond geometry (Å, °)

**Table:**

*D*—H···*A*	*D*—H	H···*A*	*D*···*A*	*D*—H···*A*
O1W—H1W···O5^v^	0.82	2.04	2.854 (4)	172
O1W—H2W···O3^vi^	0.81	1.94	2.729 (4)	165
O2W—H3W···O3^vii^	0.82	1.95	2.761 (4)	170
O3W—H6W···O4^vii^	0.81	2.02	2.802 (4)	160
O3W—H6W···O3^vii^	0.81	2.59	3.291 (4)	144
O3W—H5W···O2^iii^	0.81	1.96	2.738 (4)	161

Symmetry codes: (v) *x*−1/2, −*y*+1/2, *z*−1/2; (vi) −*x*+1/2, −*y*+1/2, −*z*; (vii) *x*, −*y*, *z*+1/2; (iii) −*x*+1/2, −*y*+1/2, −*z*+1.

## References

[bb1] Bruker (2004). *APEX2* Bruker AXS Inc., Madison, Wisconsin, USA.

[bb2] Iglesias, S., Castillo, O., Luque, A. & Romaan, P. (2003). *Inorg. Chim. Acta*, **349**, 273–278.

[bb3] Kim, J. C., Jo, H., Lough, A. J., Cho, J., Lee, U. & Pyun, S. Y. (2003). *Inorg. Chem. Commun.* **6**, 474–477.

[bb4] Moulton, B. & Zaworotko, M. J. (2001). *Chem. Rev.* **101**, 1629–1658. 10.1021/cr9900432 11709994

[bb5] Sheldrick, G. M. (2008). *Acta Cryst.* A**64**, 112–122. 10.1107/S0108767307043930 18156677

[bb6] Spek, A. L. (2003). *J. Appl. Cryst.* **36**, 7–13.

